# Evaluation of the Genericity of an Adaptive Optimal Control Approach to Optimize Membrane Filtration Systems

**DOI:** 10.3390/membranes15060157

**Published:** 2025-05-22

**Authors:** Aymen Chaaben, Fatma Ellouze, Nihel Ben Amar, Alain Rapaport, Marc Heran, Jérôme Harmand

**Affiliations:** 1 Laboratory of Environmental Biotechnology (LBE), National Research Institute for Agriculture, Food and Environment (INRAE), University of Montpellier, 34000 Montpellier, France; 2 European Institute for Membranes (IEM), University of Montpellier, 34000 Montpellier, France; marc.heran@umontpellier.fr; 3 National Institute of Applied Sciences and Technology (INSAT), University of Carthage, Tunis 1080, Tunisia; ellouze_fatma@yahoo.fr; 4 Laboratory of Modelling for Systems Analysis and Optimization (LAMSIN), National Engineering School of Tunis (ENIT), University of Tunis El Manar, Tunis 2092, Tunisia; benamar_nihel@yahoo.fr; 5 Mathematics, Informatics and Statistics for Environment and Agronomy (MISTEA), INRAE, University of Montpellier, 34000 Montpellier, France; alain.rapaport@inrae.fr

**Keywords:** ultra filtration, micro filtration, membrane fouling, membrane process optimization, model-based control, energy minimization

## Abstract

This study explores the application and robustness of an adaptive optimal control (AOC) strategy to optimize the operation of membrane filtration systems. The proposed control is based on a constant flux model where fouling is primarily due to cake layer formation. The algorithm dynamically finds the optimal ratio between the filtration (F) and backwash (BW) time ratio in response to system disturbances, thereby adapting the operational state of the membrane in order to optimize its performance in terms of energy consumption. The strategy was successfully applied to both microfiltration (MF) and ultrafiltration (UF) systems and quantitatively demonstrated its effectiveness in reducing energy consumption and controlling fouling. It proved robust against model uncertainties and demonstrated real-time adaptability even under varying and realistic disturbance conditions. The implementation of this control strategy facilitated real-time adaptation of the filtration/backwash (F/BW) ratio in response to dynamic system disturbances. The result underlines that the control behavior is predominantly driven by fluctuations in mixed liquor suspended solids (MLSSs). Compared to conventional fixed-time modes, the AOC led to significant energy savings, ranging from 7% to 30%, and membrane lifespan extension, mainly through more efficient permeate pump usage.

## 1. Introduction

Membrane filtration for wastewater treatment (WWT) is becoming more and more popular, as it offers high treatment performance and is ideally suited to water reclamation and reuse in a wide range of applications. The cost of these systems has significantly decreased in recent years; however, their operation remains relatively energy-intensive, primarily due to the persistent challenge of membrane fouling [1,2,3,4]. Membrane fouling is a fundamental limitation in membrane filtration processes, as it leads to the gradual accumulation of particles or biofilm on or within the membrane material, ultimately reducing permeability and system efficiency. This issue has been extensively addressed in the literature; for example, ref. [4] provides a comprehensive overview of membrane bioreactors (MBRs), detailing their operation, design, maintenance, and optimization. Notably, a dedicated chapter explores cost modeling and cost–benefit analysis, offering valuable perspectives for enhancing the overall performance and sustainability of MBR systems.

In fact, if the capital costs of membrane systems have significantly decreased in recent years, their operation still requires substantial energy input, mainly due to the persistent challenge of fouling. According to ref. [5], the authors modeled energy consumption in both aerobic and anaerobic MBRs (AMBRs and AnMBRs, respectively). Their findings revealed that the total energy demand in aerobic MBRs is primarily driven by the aeration required for biological processes. Regardless of the applied organic load, total energy consumption stabilizes at approximately 2 kWh·m^−3^ for wastewater with a COD concentration of 0.4 g COD·L^−1^ or higher, while AnMBRs show wider variability depending on configuration and operating conditions [6,7]. Crossflow velocity and MLSS concentration largely affect membrane fouling and pressure loss.

Although various innovative strategies, such as air scouring, relaxation, and backwashing, have been developed to improve membrane system efficiency and reduce energy consumption [4,8,9], their implementation often depends on factors such as membrane type (e.g., flat sheet, hollow fiber), suspension characteristics (e.g., viscosity, concentration of suspended solids, and Soluble Microbial Products), and the scouring method or actuators used. In ref. [10], the performance of AnMBR systems is optimized by using three statistical approaches, which allow for parameter sensitivity analysis and improve membrane fouling management. Despite their widespread use, these strategies are rarely optimized through systematic mathematical model-based approaches. Instead, they are typically designed based on empirical knowledge, operational data, or predefined procedures—commonly referred to as holistic methods—which do not rely on mathematical modeling of fouling dynamics.

A clear example is the optimization of backwashing schedules, which is usually performed through trial and error and is heavily dependent on operator experience. This limits the ability to anticipate or respond to process variations. While such heuristic or data-driven approaches are prevalent, very few can ensure true optimality with respect to a defined model and objective criterion. Most lack the ability to adapt in real time to changing operating conditions, which highlights the need for more robust model-based control strategies. Model-based approaches have gained increasing interest, particularly in wastewater treatment, where membrane systems such as microfiltration (MF) and ultrafiltration (UF) are widely employed. As highlighted in the recent review by ref. [11], many online control strategies have been proposed for membrane-based wastewater treatment; most are either heuristic or numerical in nature and do not guarantee theoretical optimality. In the context of the filtration process, the Pontryagin Maximum Principle provides the necessary conditions to determine the optimal control strategy (e.g., fouling rate, cake mass on membrane) that maximizes a performance criterion: functioning costs or water production (Table 1). It involves defining a Hamiltonian that includes both the dynamics of the production system and economic factors and then identifying the control inputs that maximize this Hamiltonian over time.

Although only a few studies have addressed optimal control strategies for membrane fouling mitigation (as shown in Table 1), they mainly focus on fixed control settings under simplified assumptions. Crucially, the availability of a validated fouling model makes it possible not only to design optimal control laws but also to assess the deviation from optimality under disturbances or uncertainties. These contributions demonstrate the potential of model-based optimization but lack adaptability to the real-time dynamics and the state of the membrane. Previously, we described the adaptive optimal control (AOC) approach, these assumptions, and application manner based on the control variables, which determine the filtration/Backwash durations to manage membrane fouling [17]. In this previous work, the AOC strategy was introduced and preliminarily validated on a single membrane filtration system under idealized conditions.

The present study aims to extend and deepen the application of this approach to more realistic scenarios. More specifically, we target membrane systems where (i) fouling is primarily due to cake layer formation and (ii) cleaning is performed through alternating sequences of filtration and physical regeneration (relaxation or backwash). Real plants are subject to disturbances, typically due to the continuous variation in the quality of the fluid to be treated, notably in MBR (concentrations of Total Suspended Solids (TSSs) or in Soluble Microbial Products (SMPs)). In addition, the process functioning parameters, such as the characteristics of the membrane or the functioning conditions of the process (as the working temperature), can also vary with time: in such a case, we must also reject such uncertainties and disturbances while optimizing the filtration. To summarize, the present study does the following:

(i) It generalizes the control approach to multiple membrane systems;
(ii) It tests it under severe disturbance and uncertainty conditions;
(iii) It quantitatively assesses its performance in terms of fouling control, energy savings, and membrane lifespan extension.

The paper is organized as follows. In the materials and methods section, both the class of models and the adaptive optimal control are detailed. In the results and discussion section, preliminary results demonstrating the interest of the proposed approach to deal with a large class of process uncertainty and disturbances are presented. Finally, some conclusions and perspectives are drawn.

## 2. Materials and Methods

The adaptive approach proposed in this paper consists of iteratively applying the optimal control approach initially proposed in Aichouche et al. (2020) [15]. This strategy was based on the hypothesis that (i) the membrane fouling is mainly due to the formation of a cake onto the membrane and (ii) the matter attached onto the membrane may be removed by backwash/relaxation. For such systems, the optimal control, allowing us to reduce the energy consumption of the permeate pump, was proposed as the computation of the optimal value of the ratio between the filtration and the cleaning time periods. Hereafter, we consider cleaning either by backwash or relaxation, the difference only being seen in the dynamic at which the matter detaches from the membrane. Thus, in the following, the terms backwash or relaxation are used interchangeably. Such an approach necessitates the use of a model of the fouling dynamics, which is recalled below.

### 2.1. Control Model

#### 2.1.1. Fouling Dynamics

In order to compute the optimal control, a model of the fouling dynamics is necessary. It consists of the modeling of the dependency of the Trans-Membrane Pressure (TMP) and the flux (J_p_) through the membrane with a “hidden variable x”, which was interpreted in Chaaben et al. (2024) [17] as the quantity of mass attached onto the membrane and responsible for the increase in the TMP to maintain a constant flux through the membrane. By convention, u = 1 means that the system is functioning in filtration while u=−1 means that the system is functioning in backwash/relaxation mode. Under these assumptions, the dynamic of the hidden variable can be written as (Equation (1)), as follows:

(1) $$\dot{x}=\frac{1+u}{2}f_{p}\left(x\right)-\frac{1-u}{2}f_{r}\left(x\right)$$

where f_p_ and f_r_ describe the dynamic of attachment and detachment of the matter onto the membrane. In this paper, these functions have the expressions that have been proposed in Aichouche et al., (2020) [15] and are given as follows (Equations (2) and (3)):

(2) $$f_{p}\left(x\right)=-a_{p}\cdot x+b_{p}$$

(3) $$f_{r}\left(x\right)=a_{r}\cdot x$$

where $a_{p}$ (s^−1^), $b_{p}$ (kg·m^−2^·s^−1^), and $a_{r}$ (s^−1^) are the control model parameters related to the dynamics of the mass deposited on the membrane

#### 2.1.2. Productivity and Energy Consumption

The produced water depends on the permeate flux (J_p_) and the physical cleaning flux (J_r_). Since the accumulated volume of produced water increases during the filtration phase, it decreases during the physical cleaning phase in the case of backwashing or remains constant in the case of relaxation. The dynamic of the water produced over time is given by Equation (4), as follows:

(4) $$\dot{p}=\frac{1+u}{2}J_{p}-\frac{1-u}{2}J_{r}$$

where J_p_ and J_r_ are the fluxes during filtration and backwash (>0)/relaxation (=0), respectively, and they are constant during the operation time. Thus, the volume of water produced from the initial time up to time t is calculated in Equation (5) as follows:

(5) $$V_{T}\left(t,u(t)\right)=\int_{0}^{t}\left(\frac{1+u}{2}J_{p}+\frac{1-u}{2}J_{r}\right)dt$$

It was further assumed that the total energy demand over a filtration period t_f_ is the sum of the energy needed during the filtration phase and the one required during the backwash or relaxation phase; this energy, denoted E_T_, depends on x(t) and is expressed by (Equation (6)), as follows:

(6) $$E_{T}\left(x(t),u\right)=\int_{0}^{t}\left(\frac{1+u}{2}E_{p}\left(x(t)\right)+\frac{1-u}{2}E_{r}\left(x(t)\right)\right)dt$$

where x(t) is the membrane state, E_p_ and E_r_ are, respectively, the required pumping energy during filtration and backwash/relaxation phases, and they can be directly calculated (by experimental data) in Equation (7).

(7) $$E_{p}=Q_{p}\cdot {TMP}_{p}\;\;\;and\;\;E_{r}=Q_{r}\cdot {TMP}_{r}$$

Also, Ep  and Er can be modeled by specific expression that depend on the deposited mass (x(t)) (simulated data). In this paper, these functions are chosen as those proposed in [15] and are expressed in Equations (8) and (9).

(8) $$l_{p}\left(x(t)\right)=c_{p}\cdot x+d_{p}$$

(9) $$l_{r}\left(x(t)\right)=c_{r}\cdot x+d_{r}$$

where $c_{p}$ (m^5^·Pa·s^−1^·kg^−1^), $d_{p}$ (m^2^·Pa·s^−1^), $c_{r}$ (m^5^·Pa·s^−1^·kg^−1^), and $d_{r}$ (m^2^·Pa·s^−1^) are hereafter called the control model parameters.

Additionally, a Performance Index (PI) was calculated (Equation (10)) to evaluate the performance of control strategies. This index determines the energy consumed by the permeate pump (W·s or W·h) relative to the volume of produced water (m³) from the initial time to time *t*.

(10) $$PI\left(t\right)=\frac{E_{T}\left(t,u\right)}{V_{T}\left(t,u\right)}=\frac{\int_{0}^{t}(\frac{1+u}{2}E_{p}\left(x(t)\right)+\frac{1-u}{2}E_{r}\left(x(t)\right))dt}{\int_{0}^{t}\left(\frac{1+u}{2}J_{p}+\frac{1-u}{2}J_{r}\right)dt}$$

### 2.2. Control Strategies: An Optimal Control Approach

Under appropriate general hypotheses and using the Pontryagin Maximum Principle, Aichouche et al. (2020) [15] analytically solved the problem of minimizing the total energy E_T_ to attain a given quantity of water treated at the free final time t_f_ in computing the best sequence of filtration and backwash phases and their optimal lengths for the specific functions f_p_ and f_r_ given by Equations (2) and (3). In particular, depending on the initial conditions, they demonstrated that the optimal control consists of alternating filtration–backwash/relaxation sequences of specific time lengths, which only depend on parameters of the control model and of the objective function chosen. While Appendix A.1 presents the determination and the practical implementation of the optimal control (singular mass ($\bar{m}$) and singular control ($\bar{u}$)), the following section provides an overview of this strategy.

#### 2.2.1. An Optimal Control Strategy and Its Limits

**Figure 1:**
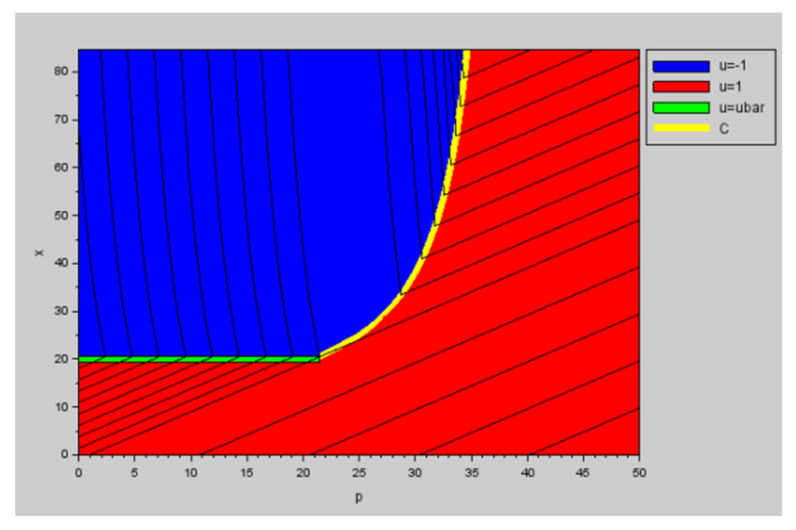
Optimal synthesis for the considered parameters in the (p, x) plane, with p being the adjoint state of the optimal control. The singular arc is in green and the switching curve in yellow, from [15].

Under its original form, Aichouche et al. (2020) applied the optimal control in open-loop for an undisturbed system [15]. The theoretical control solution is represented in Figure 1. Depending on the initial state of the membrane, this strategy consists of applying a filtration or physical cleaning phase (backwash/relaxation) to reach the singular arc defined by the singular mass ($\bar{m}$), where the singular control ($\bar{u}$) is applied until the predefined target is attained.

As long as the filtration process is submitted to constant inputs, the functions f_p_ and f_r_ only depend on constant values. However, when the system is submitted to input varying effluent characteristics, the functions f_p_ and f_r_ will have parameters that vary with time. Notably, the solution of the optimal problem depends on these parameters: thus, if the system is submitted to unknown disturbances, the solution—the optimal ratio of filtration and backwash time periods—will also change with time and it is necessary to re-compute it regularly. In other words, it is necessary to switch from a strategy where the actual state of the system is ignored to a strategy in which the actual state of the system is considered. Such an adaptive control is presented in the next section.

#### 2.2.2. An Adaptive Optimal Control Approach

The proposed adaptive optimal control strategy enables the system to dynamically adapt to input variations, ensuring “sub-optimal” performance despite the uncertainty and variability in operational conditions.

In contrast to the strategy outlined in the previous section, the adaptive optimal control (AOC) is a “closed-loop” approach. Using a predefined process model, once the singular arc is reached, i.e., when the calculated mass (Section 2.3) is equal to singular mass (Equation (A1) in Appendix A) ($m\left(t\right)=\bar{m}$), the optimal ratio between filtration and physical cleaning (via backwashing or relaxation) duration is regularly recomputed based on the current state of the system. The adaptive optimal control algorithm operates according to the following steps:

1—Parameter Identification: The most recent system data (in this study, from the last three filtration cycles) are used to estimate the model parameters by fitting the control model to observed process behavior;

2—Computation of Singular Variables: Based on the identified parameters, the singular arc variables, namely, the optimal fouling mass ($\bar{m}$) and the optimal control ($\bar{u}$)—are computed using Equations (A1) and (A2) in Appendix A;

3—Cycle Duration Calculation: Using Equation (A3) in Appendix A and the optimal control $\bar{u}$, the algorithm calculates the optimal durations of the filtration and cleaning phases (backwash or relaxation) to be applied in the upcoming cycle.

This is a “closed-loop” algorithm, i.e., after the end of each cycle, the control process repeats itself until the end of system operation.

This control version has been developed to optimally adjust the control variable in the face of system disturbances, while evaluating its effects on energy consumption and membrane clogging as a function of time.

#### 2.2.3. Control Evaluation

Here, we will compare the three following different control strategies:

- TM for Temporized Mode. This control refers to the application of filtration and cleaning sequences on a regular basis fixed once and for all without considering any optimization problem;
- OLOC for Open Loop Optimal Control. In such a control strategy, the optimal length of the filtration and cleaning phases are computed again once and for all but are the result of an optimization procedure;
- AOC for adaptive optimal control. In such a strategy, the lengths of the filtration and cleaning phases are optimal and are recomputed on a regular basis in order to adapt the control to the actual state of the system.

**Remark** **1.** *In this paper, we use the terms open- and closed-loop controls. Because the TM and the OLOC do not account on the actual state of the system, they are called open-loop approaches. On the opposite, the AOC, although it does adapt continuously (the control parameters are computed after each filtration/cleaning cycle), may be assimilated to a closed-loop approach.*

### 2.3. Two Virtual Processes: The Membrane Filtration Simulation Models

The optimal control approach proposed by [15], which aims to minimize the energy required to treat a given quantity of water, was experimentally evaluated in [16]. However, this evaluation was conducted over a short period and did not account for any input disturbances.

This paper aims not only to evaluate the robustness of an adaptive version of this latter (in order to measure its capacities to reject input disturbances) but does also evaluate the genericity of the control model used for the control synthesis. In other words, we aim to demonstrate that this adaptive optimal control can be applied to microfiltration (MF) systems but also to ultrafiltration (UF) processes. To do so, we will first identify two distinct simulation models (one for a MF process and another one for an UF process) using data from the literature, which will then be used for control design and control evaluation.

To model fouling dynamics during filtration and physical cleaning phases (backwashing or relaxation), the two simulation models will be based on the model initially proposed in [18] considering constant permeate flux operation. In these models, fouling is primarily attributed to particle deposition forming a cake layer onto the membrane. The deposition rate of particles $\left(\frac{dm}{dt}\right)$ on the membrane can be described by the following equation (Equation (11)):

(11) $$\dot{m}=\delta \cdot Q_{out}(C_{Xi}\cdot X_{i}+C_{Si}\cdot S_{i})$$

where $\dot{m}$ is the rate at which the matter deposits onto the membrane, δ is the fouling rate, $Q_{out}$ is the outlet flow rate, and Xi and Si are the concentrations of particulate matter (MLSS) and soluble products (EPS and SMP). These concentrations are commonly used to characterize biomass and soluble fouling potential in membrane bioreactors. MLSS (Xi) typically ranges between 3 and 12 g/L in MBRs [4] and serves as an indicator of biological activity and fouling. $C_{Xi}$ and $C_{Si}$, respectively, correspond to the attachment coefficients. During the physical cleaning phase, the deposited mass on the membrane decreases according to the following dynamic (Equation (12)):

(12) $$\dot{m}=-w\cdot m$$

where w represents the efficiency of physical cleaning. This relation indicates an exponential decrease in the deposited mass as a function of the cleaning efficiency.

Chu and Li (2005) [19] found that the cake layer was unevenly distributed across the entire membrane surface A_0_. In addition, [18] considered that the filter surface is variable during operation. When considering only the fouling of the cake layer, the expression for the filtering surface becomes Equation (13):

(13) $$A(t)=\frac{A_{0}}{1+\frac{m\left(t\right)}{\sigma }}$$

where σ is a parameter to normalize units (g).

The TMP shown in Equation (14), a key indicator of filtration performance, is calculated over time using Darcy’s law, as follows:

(14) $$TMP=J_{p}\cdot \mu \cdot R_{t}$$

where J_p_ is the permeate flux, μ is the viscosity, and R_t_ is the total resistance presented in Equation (15), which is the sum of the virgin membrane resistance (*R*_0_) and the resistance due to the cake layer (R_g_) shown in Equation (16), where R_g_ is proportional to the deposited mass (*m*) via a specific cake resistance coefficient (α) [20].

(15) $$R_{t}=R_{0}+R_{g}$$

(16) $$R_{g}=\alpha \cdot m$$

This model allows us to track the evolution of TMP over time and through filtration and cleaning cycles, providing a powerful tool for analyzing and optimizing the performance of the two considered MF and UF membrane bioreactors.

## 3. Results and Discussion

### 3.1. Identification of Simulation Models

#### 3.1.1. Literature Data Used to Validate the Process Simulation Model

In order to validate the genericity of our control model, we need to demonstrate its applicability to various membrane filtration systems. Data used for identifying the two simulation models under interest are taken from Gautam et al. (2022) [21], denoted system A hereafter, and data from Jeong et al. (2018) [22], denoted system B hereafter. The characteristics and operating conditions of the two systems studied are reported in Table 2 and Table 3, respectively. They were chosen because both systems are very different. The system A is a hollow fiber membrane (microfiltration) operated with filtration and backwash cycles while the process B is a flat sheet membrane operated with filtration and relaxation cycles.

#### 3.1.2. Identification of Simulation Model Parameters

**Figure 2:**
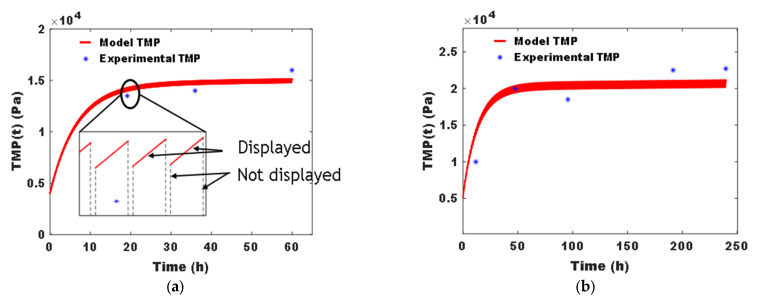
Model fitting the PTM using available data for the identification of simulation models A and B; data from [21] for system A (**a**) and [22] for system B (**b**).

To obtain simulation models that will be used for control evaluation, we identified the simulation model parameters of the models described in Section 2.3 using the data from systems A and B. More specifically, the parameters were calibrated using a classical least squares method implemented under the MATLAB-R2020a programming software. Their optimal values are reported in Table 4, and their corresponding fitting curves are shown in Figure 2.

### 3.2. Comparison of Different Control Laws Applied to Systems A and B

In order to evaluate the performances of the new adaptive control on both systems A and B in the presence of disturbances and unknown inputs, the three different control strategies presented in Section 2.2.3 were implemented in simulation and compared.

#### 3.2.1. Perturbation Description

To evaluate the response of the AOC to system disturbances, a specific sequence of perturbations was applied to both systems A and B.

**Figure 3:**
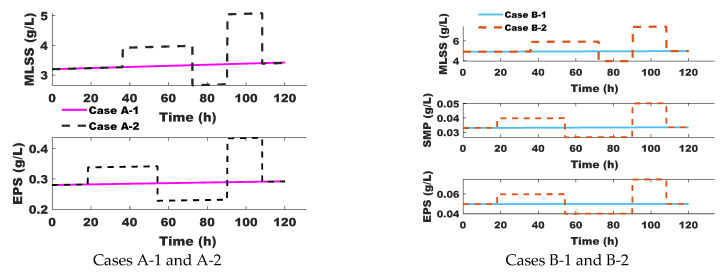
Disturbance sequence for particulate (Xi = MLSS) and soluble (Si = EPS and/or SMP) matter concentrations.

In this study, the disturbances correspond to variations in the concentrations of Mixed Liquor Suspended Solids (MLSSs) as particulate matter (X_i_) and the concentrations of Extracellular Polymeric Substances (EPSs) and/or Soluble Microbial Products (SMPs) as soluble matter (S_i_), introduced as step changes applied synchronously every 18 h. Additionally, these perturbations are illustrated in Figure 3, where it can be observed that the concentrations of soluble and particulate matter were first modified with a time delay and then simultaneously. This time shift was designed to analyze the sensitivity of the control system to different concentrations and assess its adaptability to variations in both directions: increase (positive perturbation) and decrease (negative perturbation). Finally, different step change amplitudes were applied to study the impact of varying perturbation intensities on the performance of the approach. The selected concentration ranges (MLSS: 3–5 g/L and 4.5–6.5 g/L; EPS: 0.3–0.45 g/L and 0.045–0.07 g/L) are consistent with values reported in the literature [4,23,24] and are therefore considered representative for simulation purposes.

#### 3.2.2. Control Evaluation Scenarios and Simulation Results

To evaluate the performances of the different control strategies mentioned in the Section 2.2.3, two different operating conditions were considered and applied to both simulation models. The first condition is an undisturbed operation while the second consists in a sequence of disturbances on both inputs, Xi and Si concentrations.

The results are reported as four “case studies” for all the three control strategies considered:

#Case A-1 [21]: An operation cycle consisting of 13.5 min of filtration and 1.5 min of backwash to preserve membrane permeability (with J_r_ = 1.5 J_p_) was considered for the TM strategy (Total cycle duration T_cyc_ = 15 min). It was compared with the results obtained with the two other strategies.

#Case A-2: The same operation as in case A-1 but during the operation time; the concentrations of fouling substances were disturbed with the sequence in Section 3.2.1. Again, the results were compared to those obtained with the two other control strategies.

#Case B-1 [22]: An operation consisting of 4 min of filtration and 1 min of relaxation (Tcyc = 5 min) to preserve membrane permeability was applied and compared with the two other control strategies.

#Case B-2: The same process as in case B-1 was applied but the concentrations of fouling substances were perturbed using the same disturbance sequences as in case A-2. Again, the results were compared to those obtained with the two other control strategies.

The parameters of the AOC and of the OLOC are fixed once and are given hereafter. In theory, to compute the solution of the AOC and of the OLOC, it is necessary to know the quantity of water to be treated (remember that the optimization time horizon is free, cf. Aichouche et al. (2020) [15]), which is the target of the control over the free control horizon. However, it is only used to know when leaving the singular arc over this time horizon (cf. [15]): following the way the algorithm is implemented in its adaptive form (cf. Section 2.2), in practice it is not necessary. Regarding the time horizon considered for identifying the control model parameters in the OLOC (or re-identifying it at each step for the AOC), it has been arbitrarily fixed at three cycles for both strategies as suggested in [17]. Optimally choosing this time horizon would necessitate further studies. Notice that this procedure of the (re)-identification of model parameters can only be applied if the control model is identifiable. Before implementing the identification procedure, it was checked that it is indeed the case. It means that from a set of available data (over three cycles time horizon), a unique set of optimized parameters is found with the least squares algorithm.

**Figure 4:**
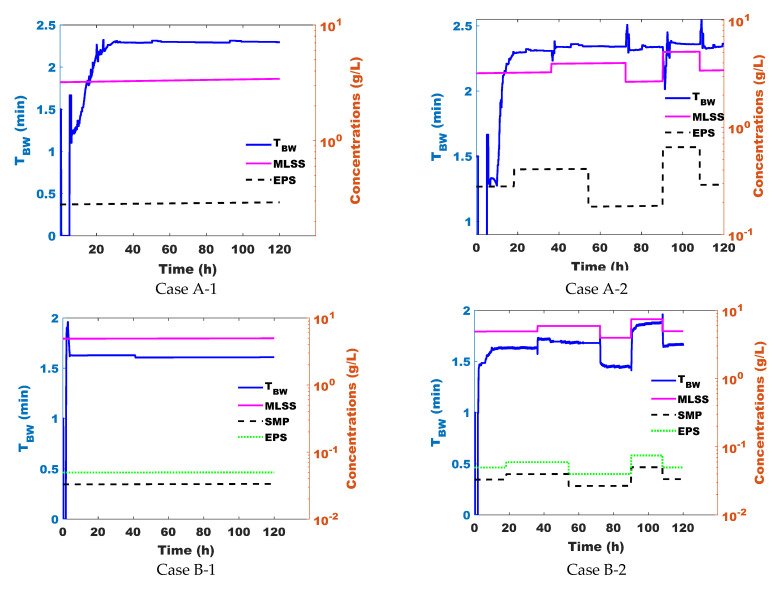
Variation in the control variable over time (time of physical cleaning—backwash or relaxation time period—phase) of the new approach in Case A-1, Case A-2, Case B-1, and Case B-2.

When comparing the results obtained with the different control strategies, the best results were obtained with the AOC strategy. The ratios of filtration/backwash for system A (or filtration/relaxation for system B) corresponding to the application of the new control law for the four case studies are plotted in Figure 4.

**Figure 5:**
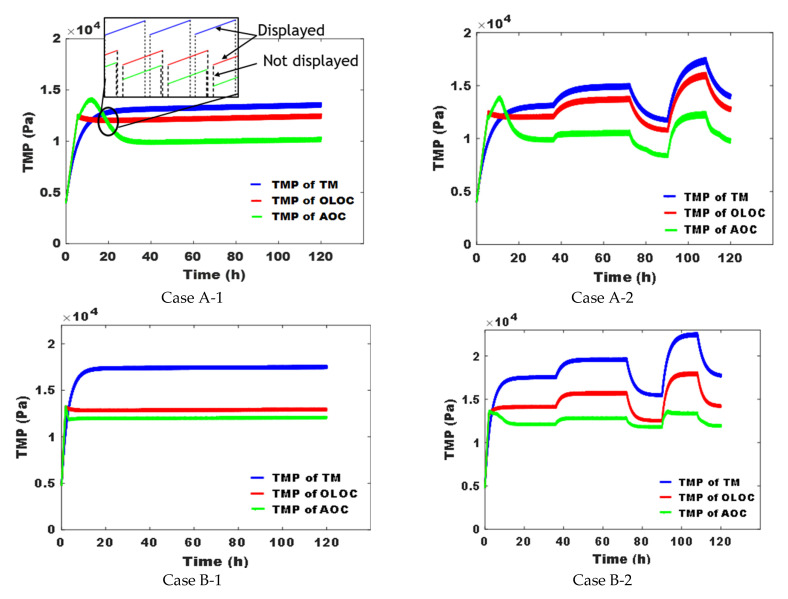
Variation in TransMembrane Pressure (TMP) over time for the 4 cases controlled by the 3 strategies.

The TMPs simulated for all case studies and all control strategies as well as the parameters of the two control models of systems A and B identified over time are plotted in Figure 5.

The results presented in Figure 4 and Figure 5 were obtained in applying the control defined as the ratio between the filtration over the backwash/relaxation time periods: for the two optimal controls, these successive control inputs are the results of the computations of the optimal control laws, which depend on the control model parameters identified at the end of each cycle. Because inputs vary with time, the control model parameters also vary with time.

In addition, the important simulation model variables, which are the mass of matter attached onto the membrane, the membrane resistances, and the membrane free surface, are reported for all cases in Appendix B.2 for the different strategies.

#### 3.2.3. Discussions and Control Performance Assessment

The optimal control strategy is based on controlling the mass deposited on the membrane while keeping energy consumption at a minimum. As explained in Section 2.2, depending on the initial state of the membrane, we proceed either in filtration or in backwash/relaxation until the singular arc $\bar{m}$ is attained [15]. In our cases at t = 0 s, the membrane is considered to be clean. Therefore, we started with cycles controlled by “u_0_ = 1” until $m=\bar{m}$ where the AOC is then turned on (and the process controlled in applying ${\bar{u}}_{i}$, the expression of which is recalled in Appendix A.1).

In Figure 4, for cases A-1 and A-2, the singular arc is attained after 5 h of operation. Thus, the duration of physical cleaning starts to increase rapidly until it stabilizes after around 35 h, which indicates that the AOC approach takes into account the state of the membrane after each cycle and responds by increasing the frequency of cleaning phases. However, for cases B-1 and B-2 (Figure 4), control over the singular arc was initiated after only 1.5 h. This difference between cases A-(1 and 2) and B-(1 and 2) could be attributed to the membrane fouling rate, which is higher for UF cases (B-1 and B-2), as well as the efficiency of physical cleaning. Indeed, relaxation is less effective compared to backwashing due to the absence of a counter-current flow, which further mitigates the fouling phenomenon. This is why the systems in cases A-1 and A-2, proceeding with backwashing as physical cleaning, took longer to reach the singular arc state. Furthermore, looking at Figure 4 (case A-2), where the disturbances of X_i_ and S_i_ concentrations were applied, we observed an instantaneous response from this control system by varying the duration of physical cleaning (by increasing X_i_ and S_i_ concentrations, we were able to reinforce the clogging phenomenon and vice-versa). The same tendency is observed for cases B-1 and B-2; the relaxation frequency varies when $m_{t}=\bar{m}$ and further changes after disturbing input concentrations. Then, reasoning on the response of the control system and the study of [17], this approach adapts to the abrupt changes in inputs. However, the response of the AOC to disturbances for case A-2 is less significant than that for case B-2 in terms of physical cleaning frequency.

Recalling that optimization in this study is performed in free time, the differences observed between the systems may be attributed to the intrinsic characteristics of the physical cleaning strategies implemented. In the case of backwash (cases A-1 and A-2), the optimal control is determined by balancing two conflicting objectives: on the one hand, increasing the duration and/or frequency of backwash sequences contributes to fouling mitigation and membrane protection; on the other hand, these same sequences require a counter-current flow that interrupts filtration and consumes additional energy, which negatively impacts system productivity. Thus, the controller must find a compromise between sufficient cleaning to limit irreversible fouling and minimizing the loss of treated volume and energy associated with frequent or prolonged backwashing. In contrast, in the case of relaxation (cases B-1 and B-2), the situation is different. Although the same optimization objectives are considered—fouling mitigation and productivity—they are no longer in direct opposition. Relaxation consists of pausing the filtration without requiring additional flow or significant energy input. Since there is no permeate flow during this phase (i.e., Jr = 0), it does not significantly penalize productivity in terms of energy costs. Therefore, the control strategy can adjust the frequency of relaxation more freely to mitigate fouling without strongly compromising energy efficiency or throughput. This fundamental difference explains why the adaptive control behaves differently depending on the physical cleaning strategy used.

In Section 3.1.2, by modeling the experimental data, the attachment coefficients of suspended solids to the membrane were found to be higher than those of soluble matter (Table 4). Furthermore, applying the sequence of disturbances by alternating between MLSS and soluble matter (Section 3.2.1) allowed us to test the sensitivity of the control to these disturbances. As a result, Figure 4 (Case A-1 and Case B-2) shows that the AOC control is more sensitive to changes in MLSS than to variations in soluble matter.

In agreement with the studies by [25,26,27], fouling rate increases with higher MLSS concentrations (higher MLSS = higher fouling). The AOC effectively adjusts the control strategy in response to MLSS concentration disturbances (Figure 4).

**Figure 6:**
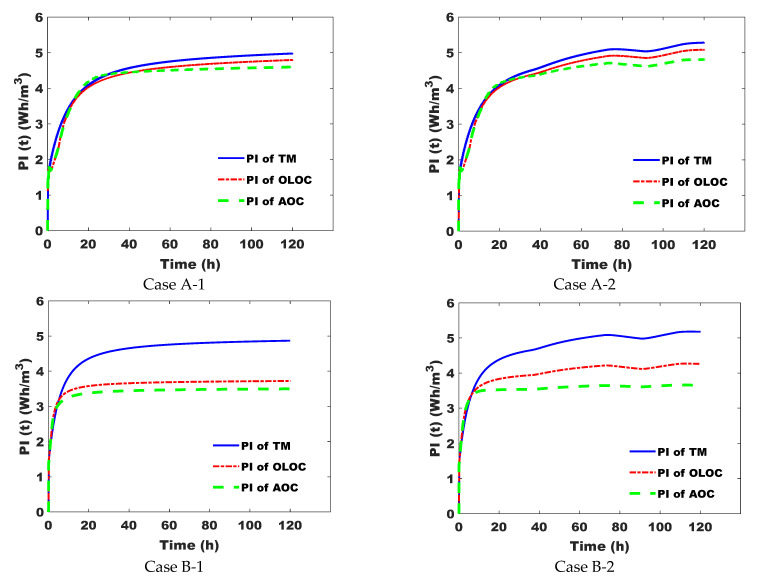
Variations in Performance Index (PI) over time, applying the 3 control strategies.

Figure 6 shows the variation in Performance Index (PI) over time for the three control strategies. For the three control strategies, their Performance Indexes increase progressively toward a kind of steady state. On the one hand, for cases A-1 and A-2, we start to observe the difference between the three types of control after about 40 h of operation. On the other hand, for cases B-1 and B-2, the difference was observed after about 5 h of operation, which explains why the optimal control approach, either applied in open or in closed loop, acts differently depending on the system and its operating mode.

#CaseA-1: The OLOC enabled us to save about 3.6% of total energy consumed/total water quantity produced (Wh/m^3^) compared to TM. In addition, our AOC approach enabled us to save 4.14% of energy consumption/water quantity produced (Wh/m^3^) compared to the OLOC strategy.

#CaseA-2: During 5 days of system operation, the application of the OLOC and the AOC allowed us to preserve energy by decreasing the Performance Index (PI) by 3.77% and 8.97%, respectively, compared to the process controlled by the TM. Comparing this case study with the previous one, the energy saving in this case following the application of our approach is more important (Figure 7 and Figure 8). Consequently, we can deduce that, considering that the only difference between the two cases is the presence or absence of abrupt changes in X_i_ and S_i_ concentrations, AOC preserves more energy compared to the other two types of control, and it preserves even more relative to the other two when the inputs are varied [17]. 

#CaseB-1: Based on the Performance Index, the AOC strategy enabled us to reduce the index by 28.2% and 6% compared with the TM control strategy and the OLOC strategy, respectively.

#CaseB-2: In this case study, the percentage reduction in the Performance Index (=percentage of energy savings) of the AOC-controlled system is greater than in case B-1 (Figure 8). Remembering that the difference between the two cases is the perturbations in case B-2, the AOC strategy makes the filtration system effectively adaptable to changes in process inputs.

The difference in the response of the two optimal control strategies (OLOC and AOC) between cases A-(1 and 2) and cases B-(1 and 2) was remarkable. In fact, for cases where backwashing is used as physical cleaning (cases A-1 and A-2), the energy gain per unit of volume is less than 10%. On the other hand, for cases where relaxation is used (cases B-1 and B-2), the energy gain is around 31 ± 6%. The observed energy savings are linked to the specific characteristics of each controlled system. In particular, the type of physical cleaning plays a key role in shaping the AOC response. In cases where cleaning is performed through backwashing, the use of counter-current flow and its associated energy consumption limit the extension of backwash durations. Conversely, when relaxation is employed as the physical cleaning method, the absence of energy constraints during these phases allows for longer cleaning periods, thereby enhancing the effectiveness of the control strategy. This is why the energy gain per unit volume is more important when the physical cleaning phase is performed through relaxation.

After five days of operation, it is essential to check the condition of the fouled membrane. To assess the impact of control strategies on membrane usage, we calculated a Membrane Usage Factor (F_UM_) (Equation (17)) during operation. This factor is expressed as the ratio between the mass attached to or within the membrane and the volume of produced water.

(17) $$F_{UM}=\frac{m_{f}}{V_{T}}$$

where *m*_*f*_ represents the final mass deposited on the membrane.

In this study, the experimental values of the final mass deposited on the membrane were not determined in the works of [21,22]. However, they were estimated using the validated models presented in Section 3.1 and represented in the Appendix B.

**Figure 9:**
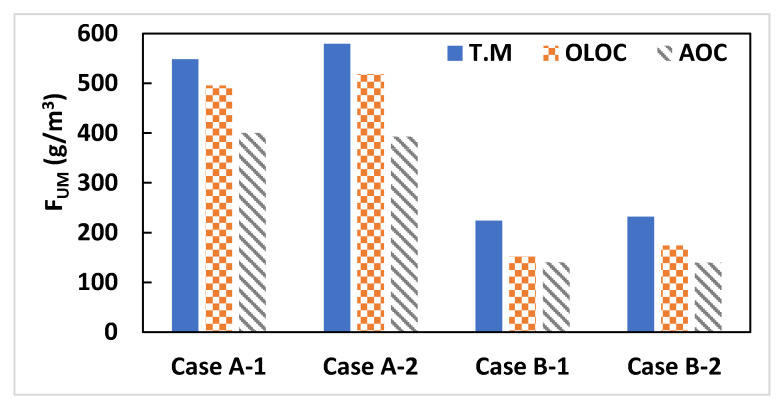
Final deposited mass of 4 cases controlled with the 3 strategies.

Figure 9 shows the F_UM_ after 5 days of system operation, indicating that in all cases, the F_UM_ of the system controlled by AOC is lower than that of the OLOC controlled system, and the F_UM_ of the latter is lower than the one of the system controlled in TM. It may thus be concluded that, by producing a specific volume, the AOC strategy delays the chemical cleaning phase by mitigating the fouling comparing to the two open-loop control (TM and OLOC) strategies. In connection with [28,29,30,31] highlighting the detrimental effects of chemical cleaning on membrane degradation and lifespan reduction, the AOC strategy can help extend membrane longevity by decreasing the frequency of chemical cleanings. This approach not only minimizes membrane wear but also reduces maintenance costs, including those associated with cleaning agents, operational interventions, and membrane replacements.

## 4. Conclusions

In this study, the adaptive optimal control (AOC) strategy was applied and validated on two distinct membrane filtration systems (MF and UF), demonstrating its generic applicability and its ability to adapt in real time to fouling dynamics. By using a simple mathematical model, the dynamic system is described after each cycle in order to determine the optimal control variable of the next cycle. The AOC strategy achieved significant energy savings ranging from 7% to 30% and better fouling mitigation, which was the main objective of its development. In addition, the AOC approach can increase membrane longevity and reduce maintenance costs by delaying the chemical cleaning phase. In the presence of disturbances and uncertainties, the optimal control continuously adapts control parameters to the actual fouling dynamic. This makes it a robust and flexible solution for membrane operation under varying conditions. It is an important step forward since, instead of an open-loop control, the optimal control works now in a closed-loop.

In the future, we will investigate the robustness of the proposed adaptive controller with respect to different kinds of fouling mechanisms and try to develop new optimal controllers for more detailed membrane fouling models. If these preliminary results show that in all simulations, the AOC strategy does better than the others, it needs to be completed with tests using other kinds of disturbances and uncertainty (input ramps, sinusoids, multiple steps, etc.) and with other membrane filtration processes either using additional simulations involving an integrated model coupling biological and filtration components of the process or, even better, in applying these strategies on a real experimental system.

## Figures and Tables

**Figure 7:**
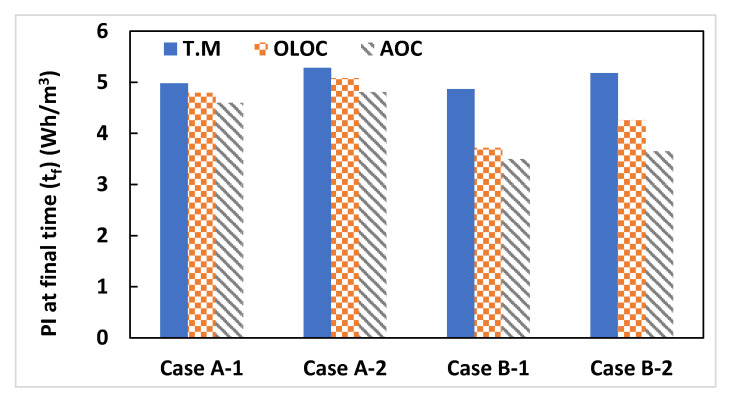
Presentation of final values (at t_f_ = 120 h) of the Performance Index (PI).

**Figure 8:**
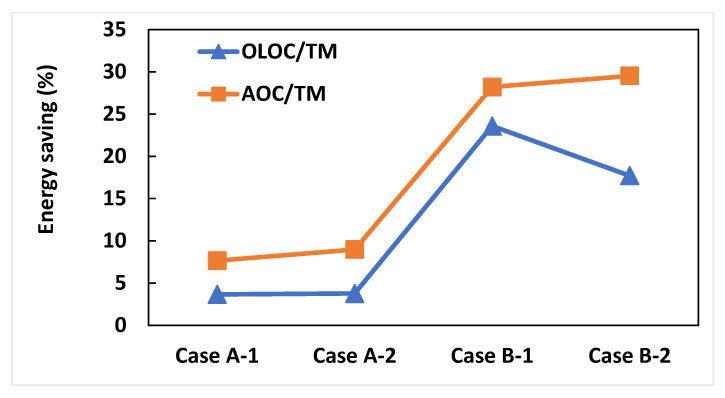
Energy saving of two optimal control strategies (OLOC and AOC) compared to TM.

**Table 1:** Various optimization targets of the optimal control approach to manage membrane fouling.

Type of Filtration System	Optimization Target	Actuators	Main Results	Reference
Dead-end filtration for raw Lake Houston water treatment	Maximize the productivity	Hydraulic backwashing period	By increasing frequency backwashing the total volume is multiplied 4 times during 10 h of operation	[12]
Batch diafiltration	Functioning costs	Inflow of the diluant and the permeate flow-rate	More than 80% reduction in the processing time is shown over the traditional operation	[13]
Unstirred, dead-end MF cell)	Maximize the productivity	Timing of hydraulic backwashes	Compared to the baseline scenarios, the net volume of water treated increased by between 17% and 61%	[14]
MF/UF–Resistance in series model	Functioning costs	Filtration/Backwash time period	Determination of an optimal feedback synthesis with minimization of energy consumption	[15]
Submerged membrane bioreactor pilot in WWTP of Charguia (Tunis, Tunisia)	Functioning costs	Filtration/Backwash time period	7% reduction in consumed hydraulic pump energy	[16]

**Table 2:** Membrane characteristics systems A and B [21,22].

Membrane Characteristics	System A	System B
Manufacturer	SINAP	Zenon Env
Membrane type/Material	Hollow fibre/PVDF	Flat sheet/PVDF
Pore size	0.16 μm	0.08 μm
Membrane surface	0.01 m^2^	0.045 m^2^
Initial membrane resistance (m^−1^)	1.45 × 10^+12^	3.33 × 10^+12^

**Table 3:** Operating conditions of systems A and B [21,22].

Membrane Characteristics	System A	System B
Qin (L·d^−1^)	2.52	4.27
HRT (d)	1.97	0.94
SRT (d)	4.63	-
Flux Jp (LMH)	6	4
V_R_ (L)	5	4

(-): Not defined.

**Table 4:** Parameters identified for systems A and B.

Parameter	Cx (s^−1^)	Cs (s^−1^)	ω (s^−1^)	σ (g)	δ (s)	α (m·g^−1^)
System A [21]	1.4 × 10^−5^	3 × 10^−7^	5 × 10^−4^	10	1.8 × 10^+5^	2 × 10^+12^
System B [22]	3 × 10^−5^	5.5 × 10^−6^	4.2 × 10^−4^	10	5.04 × 10^+4^	3 × 10^+12^

**Figure A1:**
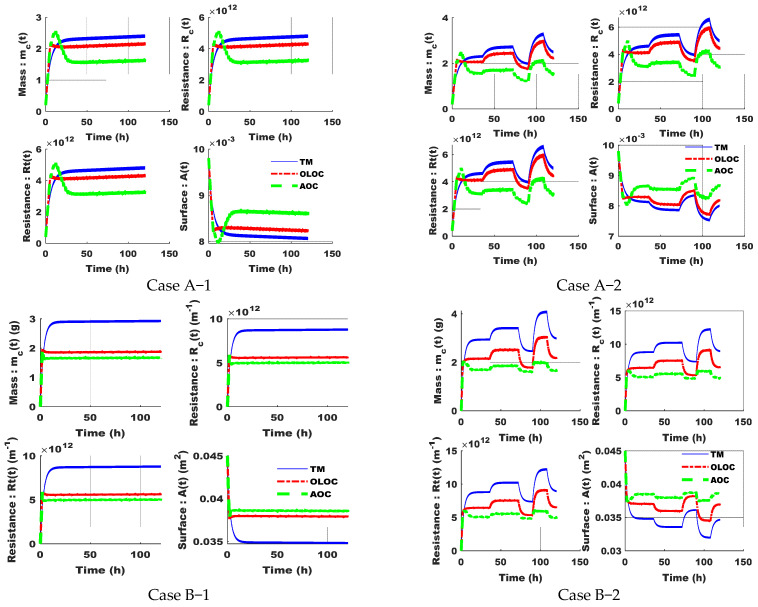
Representation of variation in the mass of matter attached onto the membrane (m_c_), the membrane resistances (R_c_ and R_t_), and the membrane free surface (A) for the different strategies.

**Figure A2:**
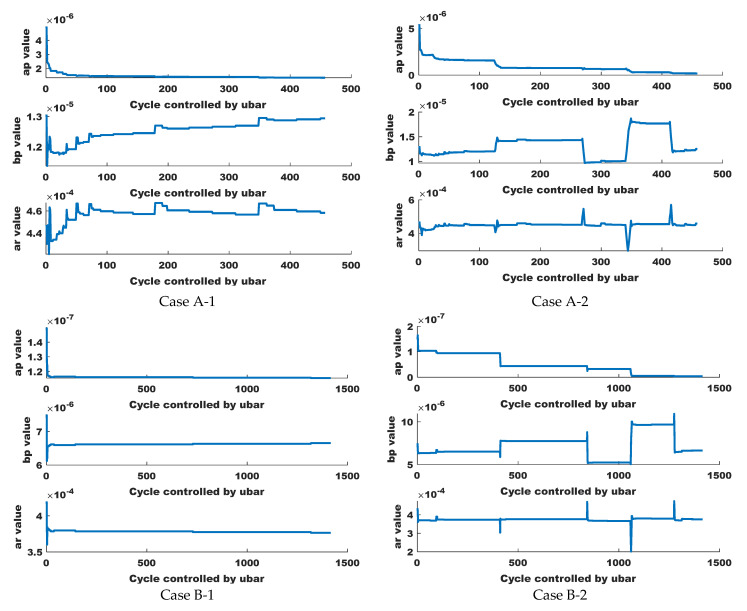
Variation in the fouling parameters in the control model (a_p_, b_p_, and a_r_) over time during the application of the AOC strategy.

**Figure A3:**
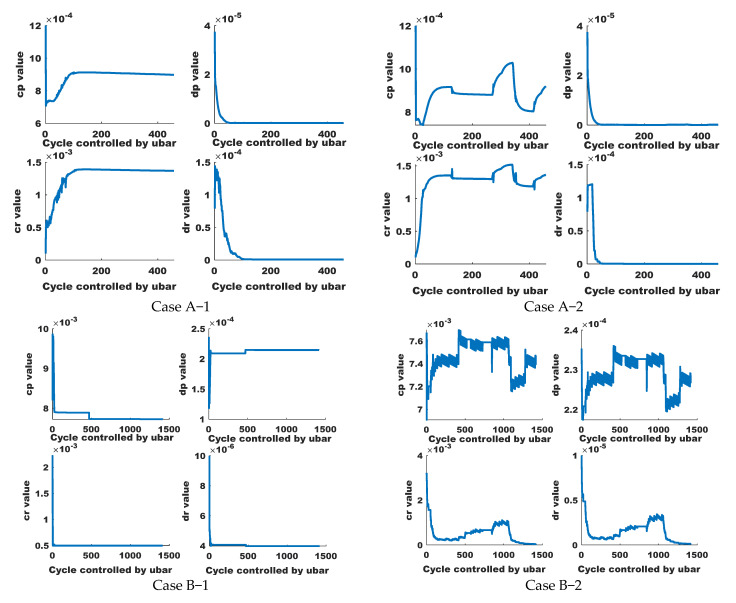
Variation in the energy consumption parameters in the control model (c_p_, d_p_, c_r_, and d_r_) over time during the application of the AOC strategy.

## Data Availability

The data used in this study are available upon request from the corresponding author.

## Abbreviations and Nomenclature

The following abbreviations and nomenclature are used in this manuscript:

**Parameter**	**Signification**
*A*_0_	Entire membrane surface (m^2^)
*AOC*	Adaptive optimal control
$a_{p}$	Model parameter related to the mass during filtration (s^−1^)
$a_{r}$	Model parameter related to physical cleaning efficiency (s^−1^)
$b_{p}$	Model parameter related to filtration (kg·m^−2^·s^−1^)
$c_{p}$	Model parameter related to the filtration energy (m^5^·Pa·s^−1^·kg^−1^)
$c_{r}$	Model parameter related to the physical cleaning energy (m^5^·Pa·s^−1^·kg^−1^)
$C_{Si}$	Attachment coefficients of soluble products (Si) (s^−1^)
$C_{Xi}$	Attachment coefficients of particulate matter (Xi) (s^−1^)
$d_{p}$	Model parameter related to the filtration energy (m^3^·Pa·s^−1^)
$d_{r}$	Model parameter related to the physical cleaning energy (m^3^·Pa·s^−1^)
*EPS*	Extracellular Polymeric Substances (g·L^−1^)
*E_T_*	Total required energy pumping (Wh)
*F_UM_*	Membrane Usage Factor (g·m^−3^)
*HRT*	Hydraulic Retention Time (d)
$J_{p}$	Permeate flux (LMH)
$J_{r}$	Physical cleaning flux (LMH)
*m* (or *x*)	Deposited mass (g)
$\dot{m}$ (or $\dot{x}$)	Rate of matter deposition onto the membrane (g·h^−1^)
*MLSS*	Mixed Liquor Suspended Solids (g·L^−1^)
$\bar{m}$	Optimal mass of cake layer (g)
*OLOC*	Open-Loop Optimal Control
*PI*	Performance Index (Wh·m^−3^)
$Q_{in}$	Inlet flow rate (L·d^−1^)
$Q_{out}$	Outlet flow rate (L·d^−1^)
*R_t_*	Total resistance (m^−1^)
*R*_0_	Virgin membrane resistance (m^−1^)
*Rg*	Cake layer (m^−1^)
*SAD*	Specific Aeration Demand (Nm³·h^−1^)
*Si*	Soluble Products (g·L^−1^)
*SMP*	Soluble Microbial Products (g·L^−1^)
*SRT*	Solids Retention Time (d)
*T_BW_*	Duration of physical cleaning phase (min)
*TM*	Temporized Mode
*TMP*	TransMembrane Pressure (Pa)
u	Control variable
$\bar{u}$	Optimal/singular control variable
$V_{R}$	Reactor volume (L)
*V_T_*	Volume of net produced water (m^3^)
w	Efficiency of physical cleaning (h^−1^)
*Xi*	Particulate matter (g·L^−1^)
*α*	Specific cake resistance coefficient (m·g^−1^)
δ	Fouling rate (s)
*σ*	Parameter to normalize units (g)
μ	Viscosity (Pa·s)

## Appendix A

### Appendix A.1. Optimization Strategy

By applying the Pontryagin’s Maximum Principle (PMP) and following the optimization procedure proposed in [15], we can calculate the singular mass $\bar{m}$ (Equation (A1)), which only depends on the identified parameters of the control model (Figure A2 and Figure A3).

(A1)
$$\bar{m}=\left(b_{p}J_{r}+\frac{\sqrt{b_{p}^{2}J_{r}^{2}+\frac{a_{r}b_{p}\left(d_{p}J_{r}+d_{r}J_{p}\right)+b_{p}^{2}c_{r}J_{r}}{a_{r}c_{p}-a_{p}c_{r}}}}{a_{p}J_{r}+a_{r}J_{p}}\right)$$

Once the singular mass $\bar{m}$ is computed, we can determine the singular control $\bar{u}$ established by Aichouche et al. (2020) [15] and given by

(A2)
$$\bar{u}=\frac{-(a_{p}+a_{r})*\bar{m}+bp}{(a_{r}-a_{p})*\bar{m}+bp}$$

The singular control $\bar{u}$ has no physical meaning since it cannot take values other than 1 and −1. Therefore, we need to approximate the control $\bar{u}$ solely by filtration cycles (u = 1) and backwash/relaxation cycles (u = −1), always keeping the variation in the mass m(t) as close as $\bar{m}$ as long as we are on the singular arc. In addition, it is straightforward to compute the ratio of the filtration over the backwash time using the following equation:

(A3) $$\bar{u}=\frac{t_{F}-t_{bw}}{t_{F}+t_{bw}}$$

with t_F_ and t_bw_ being the filtration time and backwash/relaxation time, respectively, over an operating cycle.

## Appendix B

### Appendix B.1. Simulation Results—m_c_(t), R_c_(t), R_t_(t), and A(t) for the Simulated Case Studies

The mass of matter attached onto the membrane, the membrane resistances, and the membrane free surface are reported for all cases in this appendix for the different strategies.
